# DSSylation, a novel protein modification targets proteins induced by oxidative stress, and facilitates their degradation in cells

**DOI:** 10.1007/s13238-013-0018-8

**Published:** 2014-02-11

**Authors:** Yinghao Zhang, Fang-Mei Chang, Jianjun Huang, Jacob J. Junco, Shivani K. Maffi, Hannah I. Pridgen, Gabriel Catano, Hong Dang, Xiang Ding, Fuquan Yang, Dae Joon Kim, Thomas J. Slaga, Rongqiao He, Sung-Jen Wei

**Affiliations:** 1 Medical Research Division, Regional Academic Health Center, Edinburg, TX 78541 USA; 2 State Key Laboratory of Brain and Cognitive Science, Institute of Biophysics, Chinese Academy of Sciences, Beijing, 100101 China; 3 Department of Clinical Biochemistry, Xiangya Medical College, Central South University, Changsha, 410013 China; 4 Department of Pharmacology, The University of Texas Health Science Center at San Antonio, San Antonio, TX 78229 USA; 5 Department of Molecular Medicine, The University of Texas Health Science Center at San Antonio, San Antonio, TX 78229 USA; 6 Department of Medicine, The University of Texas Health Science Center at San Antonio, San Antonio, TX 78229 USA; 7 The Cancer Therapy and Research Center, The University of Texas Health Science Center at San Antonio, San Antonio, TX 78229 USA; 8 Cystic Fibrosis and Pulmonary Diseases Research and Treatment Center, School of Medicine, University of North Carolina at Chapel Hill, Chapel Hill, NC 27599 USA; 9 Laboratory of Proteomics, Institute of Biophysics, Chinese Academy of Sciences, Beijing, 100101 China

**Keywords:** DSS1, shfm1, free radicals, oxidative stress, proteasome, protein modification, DSSylation

## Abstract

Timely removal of oxidatively damaged proteins is critical for cells exposed to oxidative stresses; however, cellular mechanism for clearing oxidized proteins is not clear. Our study reveals a novel type of protein modification that may play a role in targeting oxidized proteins and remove them. In this process, DSS1 (deleted in split hand/split foot 1), an evolutionally conserved small protein, is conjugated to proteins induced by oxidative stresses *in vitro* and *in vivo*, implying oxidized proteins are DSS1 clients. A subsequent ubiquitination targeting DSS1-protein adducts has been observed, suggesting the client proteins are degraded through the ubiquitin-proteasome pathway. The DSS1 attachment to its clients is evidenced to be an enzymatic process modulated by an unidentified ATPase. We name this novel protein modification as DSSylation, in which DSS1 plays as a modifier, whose attachment may render target proteins a signature leading to their subsequent ubiquitination, thereby recruits proteasome to degrade them.

**Electronic supplementary material:**

The online version of this article (doi:10.1007/s13238-013-0018-8) contains supplementary material, which is available to authorized users.

## Introduction

In normal cells, free radicals act as signaling molecules that contribute to the maintenance of homeostasis (Dröge, 2002). They are mainly derived from oxygen (reactive oxygen species (ROS)) and nitrogen (reactive nitrogen species (RNS)), or are formed from biomolecules interacting with ROS or RNS, e.g. protein hydroperoxides (Devasagayam et al., 2004; Li and Wogan, 2005; Luperchio et al., 1996). Under normal physiological conditions, cells are equipped with mechanisms for the removal of intracellular free radicals when they begin to exceed tolerable levels (Matés, 2000). However, in cells exposed to environmental stressors, such as heat shock, chemicals, ultraviolet (UV) radiation, and ionizing radiation (IR) (Schröder and Krutmann, 2005), or in cells under certain pathophysiological conditions (Pala and Gürkan, 2008), the resulting high ROS levels overcome intracellular defenses and can cause intracellular oxidative stress. In these stressed cells, biomolecules are susceptible to damage, despite the presence of oxidant defense mechanisms that have evolved to eliminate free radicals and restore redox homeostasis (Davies, 2001). Oxidative stress has been implicated extensively in various physiological and pathological processes, including aging (Stadtman, 1992), cataract formation (Spector, 1995), neurodegenerative diseases (Multhaup et al., 1997; Browne et al., 1999; Jenner, 2003; Uttara et al., 2009), cardiovascular diseases (Heistad et al., 2009), diabetes (Baynes and Thorpe, 1999), pulmonary diseases (Walters et al., 2008), osteoporosis (Almeida et al., 2011), chronic inflammation (Weizman and Gordon, 1990), and cancer (Brown and Bicknell, 2001; Kumar et al., 2008).

Proteins are structurally more vulnerable to oxidative damage than other biomolecules due, in part, to the relatively high rate constant for their reactions with most free radicals in stressed cells (Stadtman, 1993). Oxidative modification of proteins causes a conformational change and structural unfolding, leading to a loss of protein function (Davies and Delsignore, 1987; Soto, 2003). However, proteins damaged by environmental mechanisms or intracellular free radicals (e.g. ROS) can be fragmented, undergo abnormal cross-linking, and form toxic aggregates with other damaged proteins or with normal cellular proteins (Tyedmers et al., 2010). Such events are now known to cause various neurodegenerative disorders and certain other systemic diseases (Dobson, 1999).

In cells whose redox tone is at homeostatic levels, chaperones (e.g. Hsp27, Hsp70, and Hsp90) supervise the folding of nascent proteins being produced in the endoplasmic reticulum (ER) and help detect misfolded proteins via ER stress sensor mechanisms (Hetz, 2012). These misfolded proteins are ubiquinated, whereby ubiquitin molecules are covalently attached via an E3 ubiquitin protein ligase called carboxyl terminus of Hsp70-interacting protein (CHIP), and the ubiquitinated proteins are targeted to and degraded by the ubiquitin/proteasome system (UPS) (Park et al., 2007). In addition, late molecular events for the disposal of short-lived and oxidized proteins have been shown to include the 26S (via an ubiquitin and ATP-dependent manner) or the 20S proteasome-mediated protein degradation in the cytosol and nucleus of eukaryotic cells (Hershko and Ciechanover, 1998; Voges et al., 1999; Jung and Grune, 2008; Dunlop et al., 2009). Alternatively, others have shown that oxidized proteins are coupled with chaperones and incompletely degraded in cellular lysosomes, resulting in autophagy which is associated with the formation of polymerized, nontoxic lipofuscin-like deposits in tissues (Kiffin et al., 2004; Kaushik and Cuervo, 2012). Thus, the mechanisms of intracellular protein quality controls (for the degradation of misfolded and senescent proteins) and protein oxidative damage control (for the degradation of oxidant-damaged proteins) overlap to some extent, and both include the relatively late involvement of proteasomes. However, key questions remain regarding the early molecular events in the detection of oxidatively damaged proteins. Firstly, is there a mechanism in cells discerning oxidatively damaged proteins? Secondly, how does the mechanism target oxidized proteins to the proteolytic machinery for their degradation?

Here, we identified a novel protein modification mechanism that may answer the above questions. DSS1, a small, highly acidic and eukaryotically conserved protein, plays a key role in this mechanism. The deleted in split hand/split foot 1 (*DSS1*) gene, located on chromosome 7q21.3–q22.1, was originally identified as it is missing in patients with a dominant inherited heterogeneous limb developmental disorder called ectrodactyly or split hand/split foot malformation type 1 (SHFM1) (Crackower et al., 1996). Its encoded protein DSS1 or its orthologs (e.g. Sem1 in Baker’s yeast) is now known to be involved in many important biological and cellular processes (Pick et al., 2009), such as genome stability (Marston et al., 1999; Kojic et al., 2003; Gudmundsdottir et al., 2004; Li et al., 2006), homologous recombination and DNA repair (Yang et al., 2002; Kojic et al., 2005; Krogan et al., 2004; Zhou et al., 2007; Liu et al., 2010), cellular proliferation and neoplastic transformation (Wei et al., 2003), protein degradation (Funakoshi et al., 2004; Sone et al., 2004; Wei et al., 2008), histone modification (Qin et al., 2009), and mRNA splicing, metabolism, and export (Baillat et al., 2005; Thakurta et al., 2005; Wilmes et al., 2008; Ellisdon et al., 2012). In present study, we describe a novel role of DSS1 protein as a modifier in a novel type of protein modification targeted to proteins induced by oxidative stress *in vitro* and in cells. DSS1 forms SDS-resistant adducts with these proteins, and the formation can be promoted by Fenton’s reagent (generate hydroxyl free radical) *in vitro* and in cells subjected to UV stress, and can also be suppressed by free radical scavengers, such as DTT, NAC, Vitamin C, α-lipoid acid. These results are implying that the targeted proteins by DSS1 are products of oxidation, namely oxidized proteins. The formation of DSS1-protein adducts can also be enhanced by ATP supplementation, suggesting that this novel role of DSS1 protein as a modifier tagging target proteins is processed by an unidentified ATPase. Furthermore, the proteins tagged with DSS1 are able to be further ubiquitinated, which may enable their degradation via the UPS-mediated proteolytic mechanism. Taken together, a novel protein modification mechanism existing in cell is revealed, which may discern oxidized proteins, modify them with DSS1, and lead them to degradation.

## Results

### DSS1 forms SDS-resistant adducts with cellular proteins *in vitro* which are protected by Bortezomib, a specific proteasome inhibitor

To determine whether DSS1 could form adducts with proteins, we incubated HeLa lysates with equal amounts of the biotin-labeled recombinant DSS1-V5-His protein (DSS1-biotin) (Fig. 1A) in the absence or presence of the proteasome inhibitor, Bortezomib. After incubation, the HeLa lysates treated and untreated with Bortezomib, along with pure DSS1-biotin and HeLa lysate alone as controls, were analyzed by Western blotting using streptavidin-horseradish peroxidase (HRP) conjugate. As shown in Fig. 1B, prominent protein bands representing DSS1-biotin and its oligomers were detected at approximately the multiples of 20-kDa. In addition, multiple high molecular weight protein bands whose molecular weights were distinct from DSS1 and its oligomers were more prominent when DSS1-biotin was incubated with HeLa lysates (L3) than with DSS1-biotin alone (L1) or HeLa lysate alone (L2). Importantly, the amounts of these high-molecular-weight proteins exhibited by biotin signal were significantly increased in the presence of Bortezomib, suggesting that Bortezomib prevents their degradation due to its known proteasome inhibitory activity (Fig. 1B; L4). In order to ensure that the biotin label on the initial DSS1 probe did not itself cause the formation of the protein bands, we repeated the above experiments using an *in vitro* [^35^S]-radiolabeled DSS1-myc fusion protein, employing autoradiography to detect the labeled bands. Like HeLa lysate/Bortezomib/DSS1-biotin mixtures, Fig. 1C (L5) shows that HeLa lysate containing [^35^S]-labeled DSS1-myc/Bortezomib mixtures produced multiple DSS1-associated protein bands which were SDS-resistant. If these bands were DSS1 oligomers formed by DSS1 itself, the observed bands would have had molecular weights that are multiples of the molecular weight of DSS1, however their molecular weights were not equal to those of oligomerized DSS1. We, therefore, conclude that DSS1 forms strong, SDS-resistant associations with other proteins.

**Figure 1:**
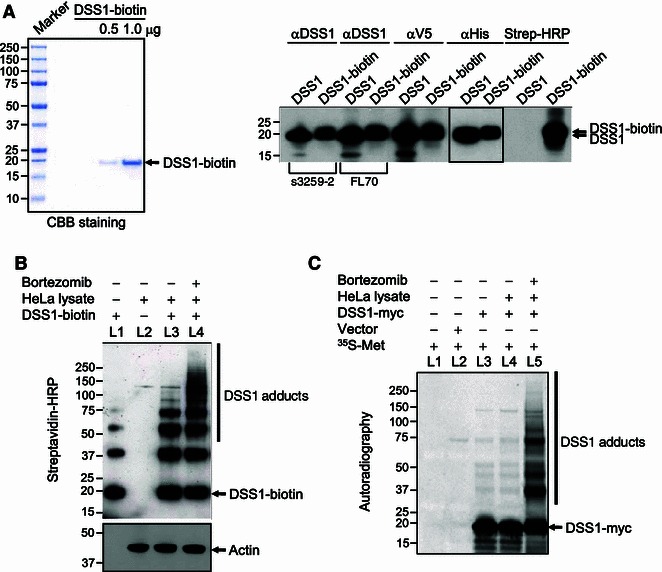
**DSS1 forms SDS-resistant adducts with proteins*in vitro*with protection of Bortezomib**. (A) After purification, the purified and/or biotin-labeled DSS1-V5-His recombinant proteins were subjected to SDS-PAGE, and analyzed by Coomassie brilliant blue (CBB) staining solution R250 or detected with WB using strepavidin-HRP or specific antibodies, including anti-DSS1s3259-2, anti-DSS1FL70, anti-V5, and anti-His. (B) After overnight incubation of DSS1-biotin (20 ng) with HeLa lysate (50 μg) at 4°C in the absence or presence of Bortezomib (20 μmol/L), the lysates were separated by SDS-PAGE and detected with streptavidin-HRP. The membrane was stripped and re-probed with an anti-actin antibody and used as a loading control. (C) The 1 μg of myc-tagged empty vector or vector with *DSS1-myc* was radiolabeled with L-[^35^S]-methionine (10 μCi) in an *in vitro* TNT cell-free protein synthesis system, and incubated overnight at 4°C with HeLa lysate in the absence or presence of Bortezomib (20 μmol/L). The lysates were prepared for IP using EZview Red anti-myc affinity resins (40 μL), followed by SDS-PAGE separation, and then the DSS1-containing bands were identified by autoradiography

### ATP promotes formation of DSS1 adducts with cellular proteins

Since attachment of several protein modifiers, such as ubiquitin and ubiquitin-like proteins, to their target proteins is an ATP-dependent enzymatic process (Hershko et al., 1980; van der Veen and Ploegh, 2012). We wondered whether DSS1 attachment to its targets is a random reaction or an enzymatic process. We first tested whether formation of DSS1-protein adducts could be regulated by ATP. To do so, the DSS1-biotin was incubated with HeLa lysates in the presence of ATP. These experiments were performed in the presence of irreversible inhibitor of cysteine peptidases*, N*-ethylmaleimide (NEM), to block the functions of ubiquitination and de-ubiquitination. Our results show that the levels of DSS1 adduct formation were significantly increased when the HeLa lysate/DSS1-biotin mixtures were supplemented with ATP (Fig. 2A; L5). In addition, the formation of DSS1 adducts was markedly increased in an ATP dose-dependent manner, when the NEM-pretreated HeLa lysates were digested beforehand with the ubiquitin specific protease 2 (USP2) to remove the pre-existing ubiquitin from its substrates (Fig. 2B; L3 to L5). This observation is noteworthy that, unlike ubiquitin and ubiquitin-like proteins, the formation of DSS1-protein adducts is not sensitive to NEM, indicating that the reaction of DSS1 tagging its target proteins does not share same mechanism with ubiquitination. Next, EDTA, a metal ion chelator frequently recruited to inhibit ATPase activity by chelating Mg^2+^ or Ca^2+^, effectively inhibited the DSS1 adduct formation (Fig. 2A and 2B; L6). Moreover, denatured HeLa lysate (marked with a pentagram star) did not exhibit an ability of forming DSS1-protein adducts in a working condition (Fig. 2A; L7). These findings strongly suggest that formation of DSS1-protein adducts is processed by an unidentified ATPase, whose catalytic mechanism is distinct from that of E1, E2, and E3 pathways performing ubiquitination (Hershko et al., 1980) and ubiquitin-like protein modifications (van der Veen and Ploegh, 2012).

**Figure 2:**
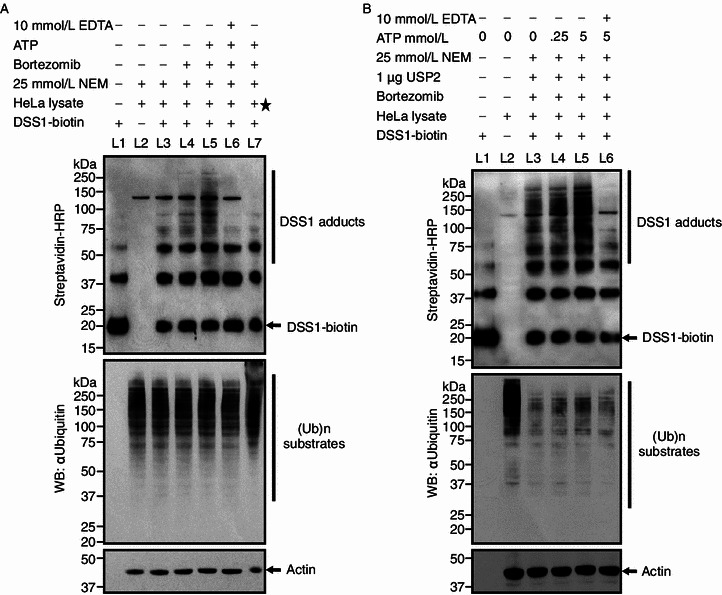
**ATP promotes the formation of DSS1 adducts with cellular proteins**. (A) After overnight incubation of DSS1-biotin (20 ng) with NEM-treated HeLa lysate (50 μg) at 4°C under the conditions indicated, the lysates were separated by SDS-PAGE and detected using streptavidin-HRP or WB with anti-ubiquitin or anti-actin antibody. The pentagram star represents that the HeLa lysate at L7 was denatured at 95°C for 10 min. ATP, 2 mmol/L; EDTA, 10 mmol/L; Bortezomib, 20 μmol/L; NEM, 25 mmol/L. (B) The manner of DSS1 adduct formation is ATP dose-dependent. The NEM-treated HeLa lysates were digested with the USP2 (1 μg) to remove the pre-existing ubiquitin from its substrates. Actin served as an equal loading control

### Free radicals increase number and protein levels of DSS1 targets

To determine what proteins are DSS1 targets, we tested multiple conditions that may regulate the formation of the tight interactions between DSS1 and its target proteins. Unexpectedly, we found that dithiothreitol (DTT) was able to reduce these interactions (Fig. S1), suggesting that DSS1 and its targets associated via the linkages of disulfide bonds. However, there is no cysteine residue in the amino acid sequence of DSS1 or in the fusion tags (e.g., V5, Myc, DDK, and HTBH) used in our experiments. Since DTT is known as an antioxidant and is able to eliminate oxidative species, such as ROS, we speculated that the observed effect of DTT was due to its elimination of oxidative species and that addition of oxidants may increase the number and level of DSS1 targets. Our data show that there was no significant increase in the levels of DSS1-protein adducts in H_2_O_2_-treated HeLa lysates (data not shown). However, when the Fenton’s reagent (a mixture of H_2_O_2_ and Fe^2+^ that can immediately generates hydroxyl free radicals OH**·**) (Fenton, 1894) was added to the HeLa lysate, more DSS1-protein adducts were detected compared with reactions lacking H_2_O_2_ and Fe^2+^ (Fig. 3; L4 and L5). This enhance of DSS1-protein adducts in the presence of H_2_O_2_ and Fe^2+^ was further augmented by the presence of ATP (Fig. 3; L5 and L7). These results suggest that free radicals, at least hydroxyl free radicals, can directly promote the increase in the number and protein levels of DSS1 targets. Since the immediate consequence of free radicals generated in cell lysates is oxidizing cellular components, including proteins, we speculate that DSS1 targets are proteins attacked by free radicals. Further more direct experimental evidence is required to confirm this.

**Figure 3:**
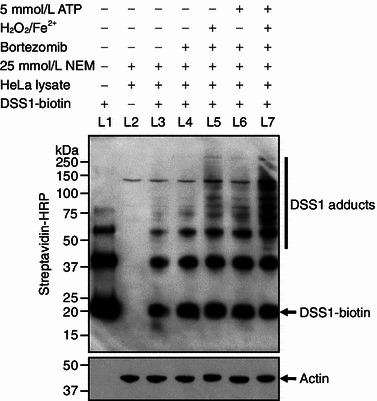
**Fenton-induced free radicals increase the number and protein level of DSS1 targets*in vitro***. Fenton-mediated oxidative stress was conducted by incubation of H_2_O_2_ and FeSO_4_ (100 μmol/L each) with NEM (25 mmol/L)-treated HeLa lysate (50 μg) for 3 h at 30°C to generate the highly reactive hydroxyl radical for oxidative damage to proteins. The DSS1-biotin (20 ng) was then incubated with the oxidant-damaged HeLa lysates (50 μg) overnight at 4°C when subjected to the conditions indicated. Actin was present as a loading control

### UV radiation causes DSS1 adduct formation in cultured cells

As UV radiation is well established as an effective means of generating ROS, in particular producing the highly reactive hydroxyl radicals OH·, in cultured cells (Masaki et al., 1995), we therefore sought to determine whether UV radiation, like chemical-induced oxidative stress, enhances the formation of DSS1 adducts in cells. To this end, we generated the stable clones using HEK293F and HeLa cell lines that were infected with retroviral vector expressing a physiological level of the HTBH-tagged DSS1 recombinant protein. The HTBH tag used contained two His×6 tags (H) that flank a protease cleavage site (T) of tobacco etch virus (TEV) and a signal peptide for endogenous biotinylation (B), making it possible to obtain highly pure protein using affinity purification of multiple steps. Upon treatment of these stable clones with UVB radiation, the whole-cell lysates were affinity purified using Ni^2+^-NTA columns and analyzed for DSS1-protein adducts using streptavidin-HRP (Fig. S2). Cells were harvested six hours later after treatment. UVB radiation led to an increase in the levels of HTBH-tagged DSS1-protein adducts in a dose-dependent manner (Figs. 4A and S3). In fact, at 6 h post-exposure to UVB radiation resulted in maximal amounts of the HTBH-tagged DSS1-protein adducts, few or no adducts remained when cells were harvested after 9–12 h (Fig. 4B), suggesting that the DSS1-protein adducts are UVB stress-inducible and are to be degraded over time.

**Figure 4:**
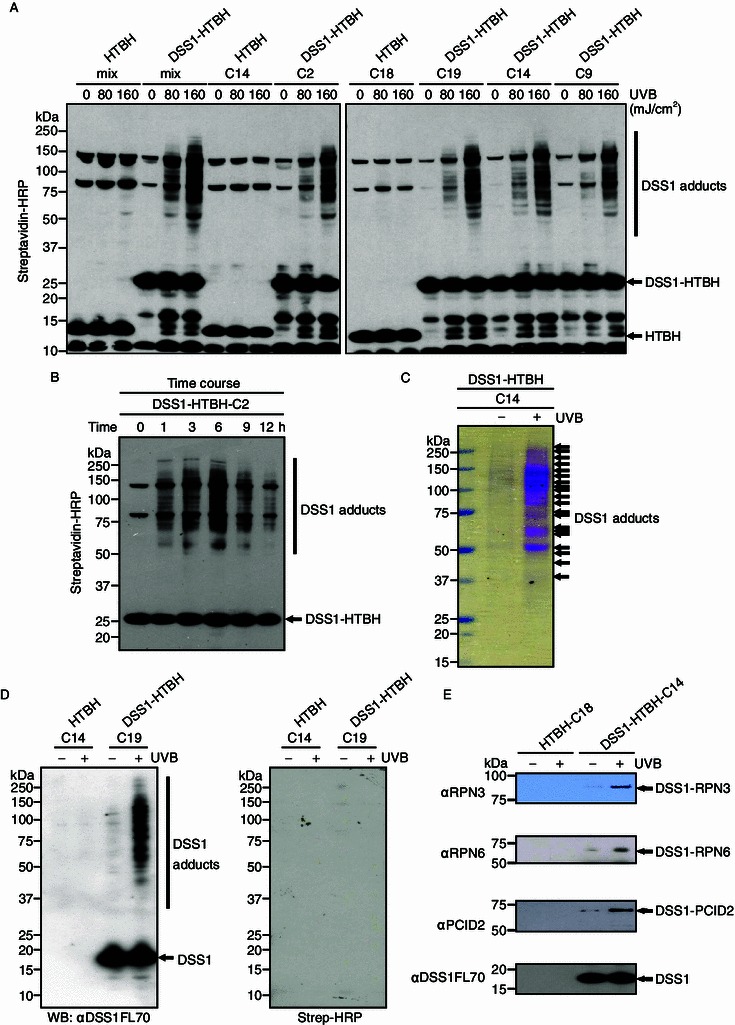
**UV radiation promotes the formation of DSS1-protein adducts in cultured cells**. (A) The formation of DSS1-protein adducts was enhanced by UV radiation in a dose-dependent manner using different stable clones. HEK293F cells stably expressing *HTBH* alone (named HTBH-mix, -C14, and -C18) or *DSS1-HTBH* (designated as DSS1-HTBH-mix, -C2, -C9, -C14, and -C19) were irradiated with or without UVB at doses ranging from 80 to 160 mJ/cm^2^. Six hours after exposure, the lysates (500 μg) were prepared for pull-down assay with Ni-NTA beads (40 μL), and DSS1 and its conjugates were identified by streptavidin-HRP. (B) DSS1-protein adducts formed in a time-dependent manner. Following exposure to UVB radiation, the HEK293F/DSS1-HTBH-C2 cells were harvested at a series of time points as indicated. The lysates (500 μg) were purified by Ni-NTA beads (40 μL), and then DSS1 and its protein adducts were detected with streptavidin-HRP. (C) DSS1-protein adducts were detected with CBB staining solution. DSS1 and its protein adducts extracted from the untreated or UVB-treated HEK293F/DSS1-HTBH-C14 cell lysates (100 mg) were pulled down by Ni^2+^-NTA (1 mL) and streptavidin-agarose beads (1 mL), cleaved with AcTEV protease, and then identified by staining with CBB-R250 solution. The potential candidates of DSS1-protein adducts were indicated by arrows. (D) DSS1 was specifically recognized in DSS1-protein adducts. The doubly affinity-purified proteins from HEK293F/HTBH-C14 or HEK293F/DSS1-HTBH-C19 cell lysates were subjected to SDS-PAGE and then analyzed by streptavidin-HRP or WB with an anti-DSS1FL70 antibody. (E) Three potential candidates of UVB-mediated DSS1-protein adducts were verified using the pull-down and WB assays. The Ni^2+^-affinity-purified proteins isolated either from the untreated or UVB (160 mJ/cm^2^)-treated HEK293F/HTBH-C18 or HEK293F/DSS1-HTBH-C14 cells were further pulled down by streptavidin-agarose beads, washed with ATP wash buffer (4×), and then cleaved in-gel by AcTEV protease to remove the HTBH tag. The DSS1 recombinant protein as well as the multiple DSS1-protein adducts were concentrated and analyzed by WB with indicated antibodies, including anti-RPN3, anti-RPN6, anti-PCID2, and anti-DSS1FL70. The membrane was stripped and re-probed with an anti-DSS1FL70 antibody and used as a positive control for the pull-down assays

Taking the advantage of having these stable cell clones expressing DSS1-HTBH or HTBH alone and the methods mentioned above of generating oxidative stress in living cells, we next tested whether free radical scavengers, such as N-acetylcysteine (NAC), Vitamin C (Vit C), α-lipoic acid (αLA) suppress the formation of DSS1-protein adducts. As expected, all of them worked effectively in a dose-dependent manner (Fig. S4). The affection of these antioxidants is consistent with that of DTT previously used *in vitro*, confirming that free radicals are the direct factors producing target proteins of DSS1.

To further demonstrate that HTBH-tagged DSS1 forms conjugates with cellular proteins in response to UVB injury, we pulled down the Ni^2+^-affinity-purified proteins from the UVB-treated DSS1-HTBH-expressing HEK293F cells using streptavidin-agarose beads, followed by washing them with SDS buffer to denature and dissociate the binding partners (e.g., proteasome subunits) from the HTBH-tagged DSS1 protein complexes (Wei et al., 2008), thus preserving only DSS1 and SDS-resistant DSS1-protein adducts (Fig. S5). The DSS1-HTBH fusion protein was then cleaved in-gel at the TEV cleavage site of the HTBH tag, thereby removing the HTBH tag and allowing the release of DSS1 and DSS1-protein adducts from agarose beads. The DSS1 and the multiple DSS1-protein adducts were detected in the doubly (two-step) affinity-purified lysates from the UVB radiation-exposed HEK293F stable cell lines expressing DSS1-HTBH using CBB-R250 protein staining solution (Fig. 4C) and an anti-DSS1 antibody (Fig. 4D; left panel). As expected, neither the DSS1 nor the DSS1-protein conjugates were detected in the doubly affinity-purified lysates of UVB-treated cells with streptavidin-HRP after removal of the biotinylated signal sequence (Fig. 4D; right panel).

To identify the potential DSS1-protein adducts induced by UVB radiation, the individual polypeptide bands, as indicated by arrows in Fig. 4C, were excised and performed with trypsinolysis and then identified by liquid chromatography coupled with tandem MS (LC-MS/MS). Only the proteins represented by at least two peptide counts and 5% amino acid coverage were considered as valid hits. We identified 39 highly potential proteins that were exclusively present in samples from the UVB radiation-exposed HEK293F/DSS1-HTBH-C14 cells and that are absent in untreated cells (Table 1). These potential candidates of the UVB radiation-induced DSS1-protein adducts are involved in many important biological events and pathways, including (1) post-translational modification, protein turnover, and chaperones; (2) translation initiation and elongation; (3) ribosome biogenesis; (4) transcriptional regulation; (5) post-transcriptional RNA processing, metabolism, and export; (6) DNA replication, recombination, and repair; (7) cytoskeleton assembly; and (8) oxidation-reduction (redox) processes. RPN3, RPN6, and PCID2 (PCI domain containing protein 2) were randomly chosen among the identified DSS1 targets for a further verification by Western blotting, to see if they are modified with DSS1 protein after cells exposure to oxidative stress induced by UVB radiation (Fig. 4E). Each of them is individually represented by their own antibody at an increased level that is about equal to the original size plus DSS1. This result also shows that the sample, extracted from UVB-treated cells, has much more DSS1-protein adducts than that from non-UVB-treated cells, which is consistent with the former results (Fig. 4C and 4D). Therefore, our data exclude the possibility that DSS1-protein adducts are oligomers formed by DSS1 itself. These results also clearly demonstrate that the attachment of DSS1 to other cellular proteins occurs in response to UVB-mediated oxidative stress.

**Table 1:** Identification of the potential candidates of UVB-induced DSS1-protein adducts by mass spectrometry

Protein name^a^	Accession #^b^	Amino acid #^c^	M.W. (Da)^d^	Reference^e^	WB^f^
**I. Post-translational modification, protein turnover and chaperones**					
26S proteasome non-ATPase regulatory subunit 11 (PSMD11/RPN6/S9)	NP_001257411	422	47,333	PCI	yes
26S proteasome non-ATPase regulatory subunit 3 (PSMD3/RPN3/S3)	NP_002800	534	60,847	PCI	yes
26S proteasome non-ATPase regulatory subunit 6 (PSMD6/RPN7/S10a)	NP_055629	389	45,400	PCI	yes
E3 SUMO-protein ligase PIAS1	NP_057250	651	71,705		
E3 ubiquitin-protein ligase tripartite motif-containing protein 33 (TRIM33) isoform α	NP_056990	1,127	122,403		
E3 ubiquitin-protein ligase tripartite motif-containing protein 33 (TRIM33) isoform β	NP_148980	1,110	120,410		
Heat shock cognate 71 kDa protein (HSP71) isoform 1	NP_006588	646	70,767		
Heat shock cognate 71 kDa protein (HSP71) isoform 2	NP_694881	493	53,387		
**II. Translation initiation and elongation**					
Eukaryotic translation initiation factor 3 subunit A (eIF3A)	NP_003741	1,382	166,439	PAM	
Eukaryotic translation initiation factor 3 subunit C (eIF3C)	NP_003743	913	105,213	PAM	yes
Eukaryotic elongation factor 1α1 (eEF1A1)	NP_001393	462	50,010		
Eukaryotic elongation factor 1α2 (eEF1A2)	NP_001949	463	50,339		
Eukaryotic elongation factor 1γ (eEF1C)	NP_001395	437	49,988		
Eukaryotic elongation factor 2 (eEF2)	NP_001952	858	95,207		
**III. Ribosome biogenesis**					
ATP-dependent RNA helicase DDX42 (RHELP)	NP_031398	938	102,844		
Midasin	NP_055426	5,596	632,692		
Ribosome production factor 2 (RPF2) homolog	NP_115570	306	35,452		
**IV. Transcriptional regulation**					
Transcription initiation factor TFIID subunit 2	NP_003175	1,199	136,855		
Transcriptional repressor protein YY1	NP_003394	414	44,582		
DNA topoisomerase 1	NP_003277	765	90,595		
Paired amphipathic helix (PAH) protein SIN3a	NP_001138829	1,273	145,045		
Staphylococcal nuclease domain-containing protein 1 (SND1)	NP_055205	910	101,866		
**V. Post-transcriptional RNA processing, metabolism and export**					
Heterogeneous nuclear ribonucleoprotein U isoform α (hnRNP Uα)	NP_114032	825	90,454		
Heterogeneous nuclear ribonucleoprotein U isoform β (hnRNP Uβ)	NP_004492	806	88,849		
Putative RNA-binding protein 15B (RBM15B/OTT3)	NP_037418	890	97,075		
RNA-binding protein 33 (RBM33)	NP_444271	1,170	129,855		
Ataxin-2 (ATX2)	NP_002964	1,313	140,152		
Serine/threonine-protein kinase pre-mRNA-processing factor 4 (PRP4) homolog	NP_003904	1,007	116,856		
WD repeat-containing protein 63 (WDR63)	NP_660155	891	102,804		
Prelamin-A/C isoform 3	NP_001033707	397	44,550		
PCI domain-containing protein 2 (THP1/PCID2 or CSN12-like)	NP_001120674	399	45,899	PCI	yes
**VI. DNA replication, recombination and repair**					
Flap endonuclease 1 (FEN1 or RAD2)	NP_004102	380	42,462		
**VII. Cytoskeleton assembly**					
Coiled-coil domain-containing protein 39 (CCDC39)	NP_852091	941	109,770		
Cytoskeleton-associated protein 5 (CKAP5) isoform α	NP_001008938	2,032	225,365		
Cytoskeleton-associated protein 5 (CKAP5) isoform β	NP_055571	1,972	218,395		
Dynein heavy chain 6, axonemal	NP_001361	4,158	475,854		
Talin-2	NP_055874	2,542	271,483		
**VIII. Redox processes**					
Thioredoxin reductase 1 (TRXR1), cytoplasmic isoform 1	NP_003321	551	60,288		
Thioredoxin reductase 1 (TRXR1), cytoplasmic isoform 3	NP_001087240	649	70,775		

^a^The proteins represented by at least two peptide counts and 5% amino acid coverage were considered only as valid hits and determined by protein score confidence index (C.I.)

^b^Accession numbers of the identified proteins were analyzed and indicated using SEQUEST against NCBI human protein database

^c^The numbers of amino acids from the potential protein candidates were indicated

^d^The molecular weight (dalton) of the identified proteins was calculated using the protein sequence relativizer

^e^PCI domain: proteasome, COP9 signalosome and eIF3; PAM domain: PCI associated module

^f^The potential candidates with PCI or PAM domain were confirmed for the formation of UVB radiation-induced DSS1-protein adducts by WB using the specific antibodies

### Four evolutionarily conserved DSS1 amino acid residues are critical for the UV radiation-induced formation of DSS1-protein adducts

Given that DSS1 is evolutionarily conserved in eukaryotic species, we next sought to determine whether its ability to form adducts with these proteins is an evolutionary-conserved function found in other eukaryotes. To address this, we fused the *HTBH-DDK* (DDK is the same with FLAG) tag DNA sequence to the 3′ end of human *DSS1* cDNA, designated as *HsDSS1-HTBH-DDK*, and used this construct to subclone DSS1 orthologous genes respectively from eight different species ranging from yeast to mice and humans, including *S. cerevisiae* (Baker’s yeast), *C. elegans* (Roundworm), *A. thaliana* (Flowering plant), *D. melanogaster* (Fruit fly), *D. rerio* (Zebrafish), *X.* *laevis* (Frog), *X.* *tropicalis* (Frog), *M. musculus* (Mouse), and *R. norvegicus* (Rat). These orthologs were transiently transfected into HEK293F cells respectively. The host cells were treated with UVB and harvested at 6 h post-exposure to UVB radiation. The formations of DSS1-ortholog adducts in these host cells were examined with streptavidin-HRP on Western blotting after a Ni^2+^ affinity purification. All DSS1 orthologs recruited in this study presented their capabilities of forming adducts with other proteins in their host cells subjected to UVB radiation (Fig. 5A). Based on this result, we speculate that the key amino acid(s) involved in connecting DSS1 to its targets must be conserved in these DSS1 orthologs. Sequence alignment of human DSS1 protein with its orthologs identified 15 amino acid residues located in human DSS1 protein that are highly evolutionarily conserved (Fig. 5B). To identify residues critical for UVB-induced formation of DSS1-protein adducts, we substituted all 15 of these highly conserved amino acids in human DSS1 protein one by one using base substitutions or point mutations. A significant decrease (~50%–70%) in the capacity of DSS1 to associate with cellular proteins, compared with wild-type DSS1, was observed when the aromatic amino acid residue W27, W39, W43, or F52 was changed into Gly (G) or Ala (A) (Fig. 5C). In contrast, there was no significant change in UVB radiation-induced formation of DSS1-protein adducts among other DSS1 mutants examined (Figs. 5C and S5). These mutants in DSS1 protein include L10A, L12A, L13A, F22L, E25G, L30A, D31R, E32G, E34G, D35R, H37I, E40G, D44G, D45G, D46G, D51G, L56I, L60I, Y65L, and G64AY65L. In addition, the formation of DSS1-protein adducts was nearly abolished when three or four of Trp (W) and/or Phe (F) were substituted with Gly (G) and/or Ala (A) at 27, 39, 43, or 52, suggesting that they are indispensable for UVB radiation-induced formation of the DSS1-protein adducts (Fig. 5D). These results were further confirmed in UVB-irradiated HEK293F/DSS1^W27GW39GW43GF52A^-HTBH stable clones when compared with HEK293F/DSS1-HTBH clones (Fig. S7). Taken together, Trp (W) at 27, 39, 43 and Phe (F) at 52 are critical residues for the formation of DSS1-protein adducts. Since these four residues are conserved in all eukaryotic species whose DSS1 gene have been sequenced, it is probable that the formation of DSS1-protein adducts in cells under oxidative stress is an evolutionarily conserved mechanism in eukaryotic species.

**Figure 5:**
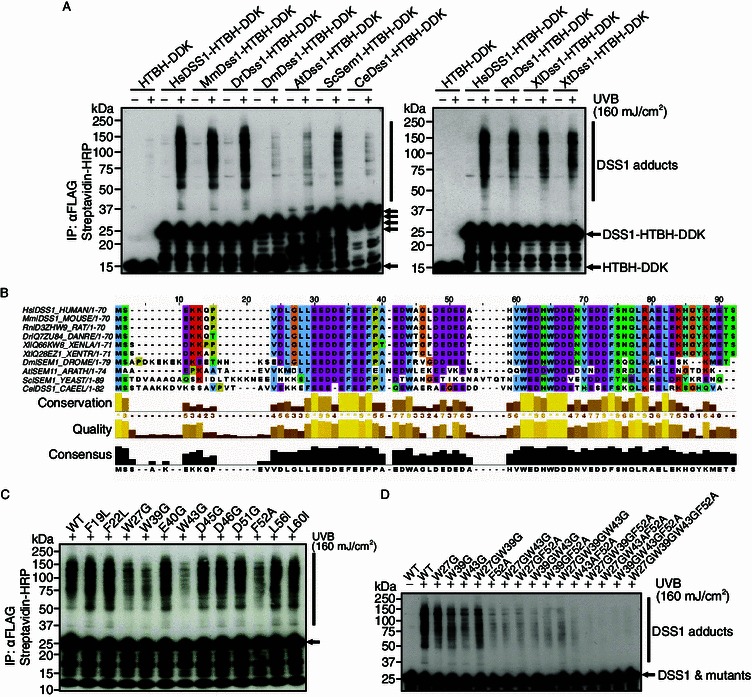
**Four evolutionarily conserved DSS1 amino acid residues are critical for the formation of DSS1-protein adducts**. (A) Formation of the DSS1-protein adducts is highly conserved through evolution. The 4 μg of *HTBH-DDK*-tagged human *DSS1* or its orthologous genes cloned from eight different species was transiently transfected into the HEK293F cells for 42 h using lipofectAMINE^®^ and PLUS^TM^ reagents. Six hours after exposure to UVB radiation at 160 mJ/cm^2^, the lysates (500 μg) were prepared for IP with EZview^TM^ Red anti-FLAG^®^ M2 affinity gel beads followed by detection with streptavidin-HRP. (B) Multiple sequence alignments for the DSS1 protein family from various species. Amino acid sequences were retrieved from Uniprot database (http://www.uniprot.org/) in FASTA format, and loaded into the Jalview program (Waterhouse et al., 2009). Protein alignments were generated using the Clustal X web services with default parameters through Jalview, and colored with Clustal X color scheme. Degree of amino acid conservation was indicated by the height and color of the “Conservation” bars. The details about the conservation, quality, and consensus calculations of DSS1 protein alignments can be found in website (http://www.jalview.org/help.html). “ - ” was introduced to optimize sequence alignments. Human: *HsDSS1* (Q13437); Mouse: *MmDss1* (Q13437); Rat: *RnDss1* (D3ZHW9); Zebrafish: *DrDss1* (Q7ZU84); Frog: *XlDss1* (Q66KW8) and *XtDss1* (Q28EZ1); Fruit fly: *DmDss1* (Q9VM46); Mouse ear cress: *AtDss1* (Q9XIR8); Baker’s yeast: *ScSem1* (O94742); Nematode: *CeDss1* (Q95Y72). (C) Identification of the amino acid residues crucial for the formation of UVB-induced DSS1-protein adducts. The site-directed mutagenesis was performed using human *DSS1* gene to substitute the highly conserved amino acid residues. The wild-type *DSS1* or its mutant genes, fused with the *HTBH-DDK* nucleotide sequences at their 3′-ends, were expressed separately in HEK293F cells. The UVB-induced DSS1-protein adduct formation was compared between wild-type DSS1 and its mutants. (D) Overexpression of the human *DSS1* gene with substitution mutations at positions W27, W39, W43, and F52 in HEK293F cells completely abrogated the UVB-induced DSS1-protein adduct formation

### Subsequent ubiquitination after DSS1-protein adducts formation

As noted above, the formation of DSS1-protein adducts, induced either by chemical or UVB-mediated oxidative stress, is increased in the presence of proteasome inhibitor, Bortezomib, implying that DSS1-protein adducts can be degraded by the proteasome. We therefore investigated whether DSS1-protein adducts are tagged with ubiquitin after their formation by incubating DSS1-biotin with NEM-treated or untreated HeLa lysates in the presence of ATP and Bortezomib, as well as increasing concentrations of Fenton’s reagent. The incubated lysates were analyzed using streptavidin-HRP to recognize DSS1-biotin and DSS1-protein adducts. As expected, Fenton’s reagent led to an increase in DSS1-protein adducts in a dose-dependent manner (Fig. 6; left top panel). A dramatic increase in the level of high molecular weight DSS1-associated proteins greater than 250 kDa was observed in NEM-deficient lysates (Fig. 6; right top panel), whereas most DSS1-protein adducts detected in lysates with NEM were smaller than 250 kDa (Fig. 6; left top panel). Furthermore, when these lysates were probed with an anti-ubiquitin antibody, ubiquitin-conjugated proteins were also of a higher molecular weight when compared with lysates treated with NEM (Fig. 6; middle panels). The data suggest that ROS-induced DSS1-protein adducts were further conjugated by poly-ubiquitin in the absence of NEM. It is worthy to notice that it is the following ubiquitination must be performed on the target proteins of DSS1, rather than DSS1 itself, since the lysine residues within DSS1 were irreversibly cross-linked with biotin. The poly-ubiquitination of DSS1-protein adducts after their formation implies that these ubiquinated DSS1-protein adducts undergo subsequent degradation mediated by the ubiquitin-proteasome system.

**Figure 6:**
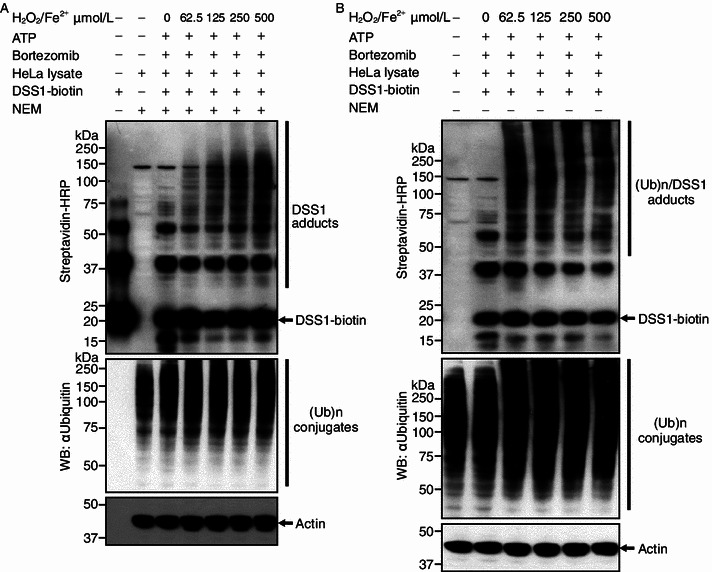
**The DSS1-protein adducts are able to be subsequently ubiquitinated**. Each reaction containing DSS1-biotin (20 ng) and NEM (25 mmol/L) (A) or non-NEM-treated HeLa lysates (50 μg total protein) (B) was incubated with increasing doses (62.5–500 μmol/L) of Fenton’s reagent in the presence of Bortezomib (20 μmol/L) and ATP (5 mmol/L). The reactions with free radicals generated by Fenton’s reagent were incubated at 30°C for 3 h. After overnight incubation at 4°C, the lysates were separated by SDS-PAGE and then followed by the detection with streptavidin-HRP or WB probed by anti-ubiquitin or anti-actin antibody. Actin served as a control for equal loading of proteins

## Discussion

The present study has revealed a cellular mechanism, in which DSS1 protein, as a novel modifier, is attached to numerous cellular proteins via an ATPase-mediated process. Also, it shows that the DSS1-protein adducts can be subsequently modified by ubiquitination, implying the degradation of these target proteins via the ubiquitin-proteasome system.

It has been observed that cellular proteins that are strongly associated with the DSS1 molecule are resistant to SDS treatment or denaturation. These associations could either arise from the interaction of covalent or non-covalent bonds. Previous studies have shown that a number of proteins could form SDS-resistant complexes with cellular proteins, including synaptic SNARE complex (Fasshauer et al., 1998), phage tail spike endorhamnosidase (Goldenberg et al., 1982), gp210 nuclear pore complex protein (Favreau et al., 2001), prion-like protein (Speransky et al., 2001), truncated mutant huntingtin exon 1 protein (Waelter et al., 2001), and ubiquitin-like protein HUB1 (Lüders et al., 2003). However, unlike the above protein complexes, the formation of DSS1-protein adduct is significantly promoted by ATP, and could be greatly suppressed by the metal ion chelator EDTA and heat-induced denaturation. These results suggest that formation of the DSS1-protein adducts is an enzymatic process catalyzed by an as yet unidentified ATPase, meaning DSS1 behaves more like ubiquitin and ubiquitin-like protein (except for HUB1) than the above proteins, whose conjugation to its target proteins is through a covalent linkage. However, unlike ubiquitin or ubiquitin-like protein modifiers, DSS1 has four critical amino acid residues, three Trp (W) and one Phe (F), involved in its attachment to the targeted proteins, implying that this conjugation is carried out by a particular mechanism (Fig. 5). For the convenience to describe this novel type of protein modification in future study, we name it as DSSylation.

It is worth considering whether the DSS1-protein adducts observed are genuine mixed species or DSS1 oligomers with unknown modifications. There are two kinds of protein post-translational modifications that could render their targets with a significant molecular weight increase, including glycation or glycosylation and ubiquitination or ubiquitin-like protein modification. However, we can rule out these modifications for the following reasons: firstly, our experimental conditions did not allow glycosylation or glycation to occur, because no sugar was supplemented *in vitro*; secondly, in the experiments supplied with NEM, the formation of the DSS1-protein adducts was not affected, whereas the ubiquitination was inhibited irreversibly (Fig. 2). Theoretically, ubiquitin and ubiquitin-like protein modification would be inhibited due to the irreversible NEM-inactivation of E1, E2, and DUBs-like enzymes. Most importantly, the proteomic identification (Table 1) and WB verification with protein specific antibodies (Fig. 4E) unambiguously demonstrated the presence of many other proteins that form adducts with DSS1.

Prior to the present study, the involvement of DSS1 protein in many critical cellular events and processes has been illustrated, but its role as a modifier attached to the proteins of interest in cells under oxidative stress has not previously been demonstrated. We show here for the first time that DSS1 plays a novel role in tagging many proteins in cells under conditions of oxidative stress. The formation of DSS1-protein adducts is conditionally induced by free radicals, which were generated, in this study, by Fenton’s reagent in cell lysate or in cells subjected to UV-induced oxidative stress, and also can be abolished by various free radical scavengers. Since the direct consequence of proteins encountered with free radicals is being oxidized, we logically infer these target proteins of DSS1 are probably oxidized proteins. This hypothesis remains to be supported by a direct demonstration. Through its attachment to these proteins, DSS1 may play a role as a common trait of the target proteins leading to the recognition of certain E3 ligase thus assists their subsequent ubiquitination and degradation via the ubiquitin-proteasome system. The putative function of DSSylation is summarized in Fig. 7.

**Figure 7:**
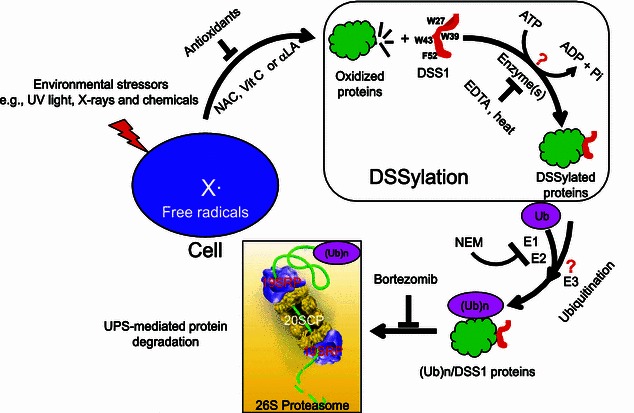
**DSSylation acts as a proposed mechanism for tagging the degradation of the oxidant-damaged proteins**. The image was adapted from the website of the United States Department of Energy’s Genomics: http://doegenomestolife.org. In stressed cells, DSSylation was promoted by free radicals (e.g., ROS) generated from environmental stressors such as UV light, X-rays, and chemicals. In addition, the UVB radiation-induced DSSylation could be suppressed after treatment of the cells with potent antioxidants, including NAC, αLA, and Vit C. Once oxidized proteins were conjugated with DSS1 molecule via an ATP-catalyzed enzymatic system, the DSSylated protein substrates were presumably able to be tagged with ubiquitin by E1/E2/E3 modification pathways followed by a recruitment of (Ub)n/DSS1 substrates into the 26S proteasome for degradation. Molecular dissection of the human DSS1 protein sequence identified four residues at positions W27, W39, W43, and F52 are required for the UVB radiation-induced DSSylation

Every living organism is frequently exposed to various oxidative stressors generated in the outer environments and inner pathological activities. Evolving a defense system against oxidative stress is critical for all creatures on earth. Scavenging mechanisms including various antioxidant reagents and enzymes have been found in cells to eliminate free radicals. However, level of free radicals often overcome the defense of scavenging mechanisms in cells exposed to environmental stressors or under certain pathophysiological conditions, resulting in oxidative stress. Proteins, the major cellular component, are naturally the major targets of free radicals. Oxidatively damaged proteins are quite cytotoxic, especially when they form aggregates. Accumulated protein aggregates have been considered as major inducing factors of multiple human diseases (Dobson, 1999). Thus, timely removal of oxidized proteins is critical for cells exposed to oxidative stress. We have shown that proteins targeted by DSS1 are enhanced in the presence of free radicals generated by Fenton’s reagent *in vitro* and by UV radiation in cultures, implying this novel mechanism introduced in this study may play a role in cleaning oxidized proteins and maintaining the viability of stressed cells. Previous studies have demonstrated that loss of the *Dss1* gene results in acute sensitivity to injures caused by oxidative stressors, such as chemicals, UV, and IR radiation (Kojic et al., 2003; Funakoshi et al., 2004). Since DSS1 is highly conserved in all eukaryotic species, we speculate that this mechanism is an ancient protective response universally conserved in eukaryotic cells.

## Materials and methods

### Reagents and antibodies

NAC, Vit C, αLA, ATP, CBB-R250, EDTA, FeSO_4_, glycerol, H_2_O_2_, imidazole, NaCl, NaH_2_PO_4_, NEM, Nonidet P40, PMSF, puromycin solution, SDS, Tris-HCl, Triton X-100, EZview^TM^ Red affinity gels (including streptavidin-HRP, anti-myc, and anti-FLAG^®^ M2), anti-actin (1:2000), and anti-FLAG M2 (1 μg/mL) antibodies were from Sigma-Aldrich (St. Luis, MO); USP2, anti-RPN7 (1:1000), and anti-ubiquitin (1:1000) antibodies from Enzo Life Sciences (Farmingdale, NY); anti-RPN6 antibody (1:1000) from Novus Biologicals (Littleton, CO); AcTEV protease, DTT, IPTG, MgCl_2_, lipofectAMINE^®^, PLUS^TM^ reagent, and anti-V5 (1:5000) antibodies from Life Technologies (Grand Island, NY); strepavidin-HRP conjugate (1:3000) from GE Healthcare (Piscataway, NJ); Bortezomib from LC Laboratories (Woburn, MA); anti-PCID2 (1:500) and anti-DSS1FL70 (1:500) antibodies from Santa Cruz Biotechnology (Santa Cruz, CA); complete EDTA-free protease inhibitor cocktail from Roche (Indianapolis, IN); anti-eIF3C antibody (1:15,000) from Bethyl Laboratories (Montgomery, TX); anti-His (1:500) antibody from EMD Biosciences (San Diego, CA); anti-RPN3 and anti-DSS1s3259-2 (1 μg/mL) antibodies from Proteintech (Chicago, IL); *Eco*RV, *Sgf*I, and *Mlu*I restriction enzymes are from New England Biolabs (Ipswich, MA).

### DNA construction and mutagenesis

The full-length human *DSS1* (*HsDSS1*) gene fused in frame with a V5 epitope-tagged sequence at the 3′ end was amplified by polymerase chain reaction (PCR). Forward and reverse primers were 5′-GGTACCA TGTCAGAGAAAAAGCAGCC-3′ and 5′-ACCGGTACGCGTAGAATCGAGA-3′, respectively. PCR was performed as described previously (Wei et al., 2003). PCR-amplified *DSS1-V5* DNA was cloned into pEXP5-CT/TOPO with a 6× His tag at its COOH-terminus (Invitrogen). *HsDSS1* and its mutant gene (*HsDSS1*^*W27GW39GW43GF52A*^) were also inserted into the *Sgf*I and *Mlu*I cloning sites of retroviral vector pQCXIP (Clontech, Mountain View, CA) with an HTBH tag at their COOH-terminals (Wang et al., 2007) (a kind gift from Dr. Lan Huang, UC Irvine, CA) using the LigaFast Rapid DNA Ligation System (Promega, Madison WI). The *HTBH* and *HsDSS1-HTBH* DNA fragments were respectively subcloned into pCMV6-entry-mycDDK plasmids (OriGene, Rockville, MD) using the *Sgf*I and *Eco*RV sites. Genes encoding DSS1 or DSS1-like proteins were also cloned from eight different species, including mouse (CD-1 skin tissue), rat (pCMV6-RnDss1-mycDDK; OriGene), frog (pDNR-LIB-XlDss1 and pCS108-XtDss1; Open Biosystems, Lafayette, CO), zebrafish (ZF4 fibroblast; ATCC), fruit fly, thale cress (Heynh. strain: S8-1-2A; ATCC), Baker’s yeast, and nematode (N2 strain). All *DSS1* genes were separately subcloned into pCMV6-HsDSS1-HTBH-DDK between *Sgf*I and *Mlu*I to replace *HsDSS1*. Deletion and point substitution mutant clones were generated using a QuikChange Site-Directed Mutagenesis Kit (Agilent, Clara, CA). All DNA sequences were verified by the DNA Core Facility (UT Health Science Center at San Antonio, San Antonio, TX) using an ABI 7700 Sequence Detection System (Applied Biosystems, Foster City, CA).

### Purification of human DSS1 recombinant protein

The *E. coli* strain *BL21* (*DE3*) was transformed with the pEXP5-CT/DSS1-V5-His plasmid encoding a human wild-type DSS1 with a V5-His tag at its COOH-terminus by a heat-shock method. The expression of DSS1-V5-His recombinant protein was induced by adding 1 mmol/L of IPTG for 3 h at 37°C with a vigorous shaking till the cell density reading at OD_600_ = 0.5–0.6. The bacterial cells were harvested by spinning at 4,000 *g* for 20 min at 4°C and re-suspended in 200 mL lysis buffer [50 mmol/L Tris-HCl (pH = 8.0), and 1 mmol/L PMSF]. After incubation for 30 min at 25°C room temperature, the cells were processed by sonication with Bioruptor (pulse 5 s on and 23 s off; total 10 min) (diagenode, Sparta, NJ) to lyse cells and shear DNA completely. The crude extract was centrifuged at 50,000 *g* for 20 min at 4°C and then flew through a 2 mL Ni-NTA nickel column (Novagen), which was pre-equilibrated with 20 mmol/L Tris-HCl (pH = 8.0), 150 mmol/L NaCl, 0.1% Triton X-100, and 40 mmol/L imidazole. After applying the sample, the column was washed with 40 mL wash buffer [20 mmol/L Tris-HCl (pH = 8.0), 500 mmol/L NaCl, and 40 mmol/L imidazole]. The DSS1-V5-His recombinant protein was eluted with 1× TBS [20 mmol/L Tris-HCl (pH = 7.4) and 0.9% NaCl] containing 50 mmol/L EDTA followed by loading it onto the 1 mL HiTrap Capto DEAE ion exchange column (GE Healthcare). The bound DSS1-V5-His protein was eluted by a linear salt gradient from 150 mmol/L to 1 mol/L NaCl. The pooled fractions containing DSS1 protein were collected and the NaCl salt concentration was adjusted to 2 mol/L. The DSS1-V5-His protein was further purified after loading onto a 1 mL HiTrap phenyl HP column (GE Healthcare). The purified DSS1-V5-His was subjected to the Superdex 75 10/300 GL size exclusion chromatography (GE Healthcare) to exchange the buffer system from Tris-HCl to 1× PBS (pH = 7.4) (137 mmol/L NaCl, 2.68 mmol/L KCl, 10.1 mmol/L Na_2_HPO_4_, and 1.76 mmol/L KH_2_PO_4_). The purified DSS1-V5-His fusion protein was collected and concentrated to 5 mg/mL. The DSS1-V5-His fusion protein was subjected to 4%–12% Bis-Tris SDS-PAGE gels (Life Technologies), verified by protein staining with CBB-R250 solution.

### DSS1 protein biotinylation

A one-step procedure for DSS1 protein biotinylation was performed by EZ-Link NHS-PEG4 kit (Pierce, Rockford, IL). The *N*-hydroxysuccinimide ester (NHS) group reacts specifically and efficiently with the side chain of Lys (K) residues and the NH_2_-terminal amino group of DSS1-V5-His fusion protein to form irreversibly stable amide bonds. The DSS1-V5-His recombinant protein was labeled for 1 h with NHS-PEG4-biotin at a molar ratio of protein/biotin = 1/40 at 25°C room temperature and then moved to 4°C for overnight incubation. The free biotin was removed by overnight dialysis at 4°C cold room using the Slide-A-Lyzer cassettes (Pierce) with 10-kDa MWCO against ice-cold 1× PBS. The biotin-labeled DSS1-V5-His was applied to SDS-PAGE and confirmed by CBB-R250 protein staining and WB with streptavidin-HRP and antibodies as indicated.

### Cell-free protein synthesis

*In vitro* TNT T7 Quick Coupled Transcription/Translation System was conducted to synthesize the [^35^S]-radiolabeled DSS1-myc fusion protein as described in the instructions (Promega). For each reaction, 40 μL TNT T7 master mix (T7 RNA polymerase, RNasin ribonuclease inhibitor, nucleotides, salt, and reticulocyte lysate), 1 μg pcDNA3.1B+/DSS1-myc-His, 2 μL L-[^35^S]-methionine (20 μCi) (PelkinElmer, Waltham, MA), and nuclease-free water were added to a final volume of 50 μL. The cell-free protein synthesis was carried out at 30°C for 1.5 h. DSS1 and its protein adducts were developed by autoradiography using a Typhoon 9410 PhosphorImager with the ImageQuant image analysis software version 5.2 (GE Healthcare).

### *In vitro* assay for DSS1 adduct formation

The total protein lysates (50 μg) extracted from cells were incubated with DSS1-biotin (20 ng) in a mass ratio of 2500:1. The assay for DSS1 conjugation to its target proteins was performed overnight at 4°C with rotation in the presence of ATP and Bortezomib (20 μmol/L). The reaction was stopped by an addition of equal volume of 2× SDS sample loading buffer followed by incubation for 10 min at 95°C. The DSS1-protein adduct assay was analyzed by SDS-PAGE and then detected with strepavidin-HRP at 1:3000.

### Cell cultures and UVB irradiation

HEK293F (Life Technologies) and RetroPack^TM^ PT-67 cell lines (Clontech) were cultured in the Dulbecco’s modified Eagle’s medium (D-MEM) supplemented with 10% fetal bovine serum (FBS), 2 mmol/L glutamine, 100 units/mL penicillin, 100 μg/mL streptomycin sulfate, and 1 mmol/L sodium pyruvate (Life Technologies). HeLa cells (ATCC) were maintained in Eagle’s minimum essential medium (E-MEM) with 10% FBS. The cell lines grown at 37°C Forma Water-Jacketed CO_2_ incubator (Thermo Fisher Scientific Inc., Waltham, MA) with a humidified 5% CO_2_/95% air were free of mycoplasma infection. HEK293F and HeLa stable cell clones or transiently transfected HEK293F cells were exposed to UVB radiation, as described previously (Kim et al., 2010). For UVB radiation studies, the USHIO G15T8E midrange lamp bulbs with a peak emission at 306 nm were used. The fluence rate was measured with an IL1400A radiometer/photometer coupled to a SEL240/UVB-1/TD detector (International Light inc., Newburyport, MA).

### Immunoprecipitation and Western blotting

The cells were washed once with 1× PBS and re-suspended in ice-cold ATP lysis buffer consisting of 50 mmol/L NaH_2_PO_4_, 100 mmol/L NaCl, 10% glycerol, 5 mmol/L ATP, 5 mmol/L MgCl_2_, 0.5% Nonidet P40, 1 mmol/L DTT, 1× protease inhibitor (EDTA-free), and 20 μmol/L proteasome inhibitor Bortezomib (Lee et al., 2010). Cells were next disrupted by using Dounce homogenizer (Wheaton, Millville, NJ) for 25 strokes and gently rocked on an orbital shaker at 4°C cold room for 15 min to lyze cells thoroughly. The total protein lysates were centrifuged at 14,000 *g* for 15 min at 4°C. The protein concentration was determined by BCA (Pierce). The proteins were pulled down by immunoprecipitation (IP), separated by SDS-PAGE, and then transferred onto the Hybond-ECL nitrocellulose membranes (GE Healthcare). The membrane was probed with the primary antibodies, detected using the HRP-conjugated secondary antibodies (1:3000) (GE Healthcare) and enhanced chemiluminescence (ECL) (GE Healthcare). The membrane was stripped and re-hybridized with anti-actin antibody as an equal loading control.

### Identification of the DSS1-protein adducts by mass spectrometry

The potential candidates of DSS1-protein adducts were identified by interrogating the MS and MS/MS, and analyzed using SEQUEST against NCBI human protein database, as described previously (Deterding et al., 2000). The MS results were filtered, sorted, and displayed using the Bioworks 3.2 at the Proteomic Core Facility Center in the Institute of Biophysics, Chinese Academy of Sciences, Beijing, China.

## Electronic supplementary material

Below is the link to the electronic supplementary material.

Supplementary material 1 (PDF 711 kb)

## Abbreviations

CBB: Coomassie brilliant blue
CHIP: carboxyl terminus of Hsp70-interacting protein
D-MEM: Dulbecco’s modified Eagle’s medium
DSS1: deleted in split hand/split foot 1
DTT: dithiothreitol
ECL: enhanced chemiluminescence
E-MEM: Eagle’s minimum essential medium
ER: endoplasmic reticulum
FBS: fetal bovine serum
HRP: horseradish peroxidase
HTBH: His×6-TEV cleavage site-biotinylated signal peptide sequence-His×6
IP: immunoprecipitation
IR: ionizing radiation
αLA: α-lipoic acid
NAC: N-acetylcysteine
NEM: *N*-ethylmaleimide
NHS: *N*-hydroxysuccinimide ester
PAM: PCI associated module
PCI: proteasome, COP9 signalosome, and eIF3
PCID2: PCI domain containing protein 2
PCR: polymerase chain reaction
redox: oxidation-reduction
RNS: reactive nitrogen species
ROS: reactive oxygen species
SHFM1: split hand/split foot malformation type 1
TEV: tobacco etch virus
UPS: ubiquitin/proteasome system
USP2: ubiquitin specific protease 2
UV: ultraviolet
Vit C: Vitamin C
WB: Western blotting
