# Supplementary material for: DSSylation, a novel protein modification targets proteins induced by oxidative stress, and facilitates their degradation in cells
Source: Protein Cell. 2014 Feb 11;5(2):124–40. doi: 10.1007/s13238-013-0018-8 (PMC3956975; doi:10.1007/s13238-013-0018-8)
Supplement: Supplementary file 1 [file 13238_2013_18_MOESM1_ESM.pdf]

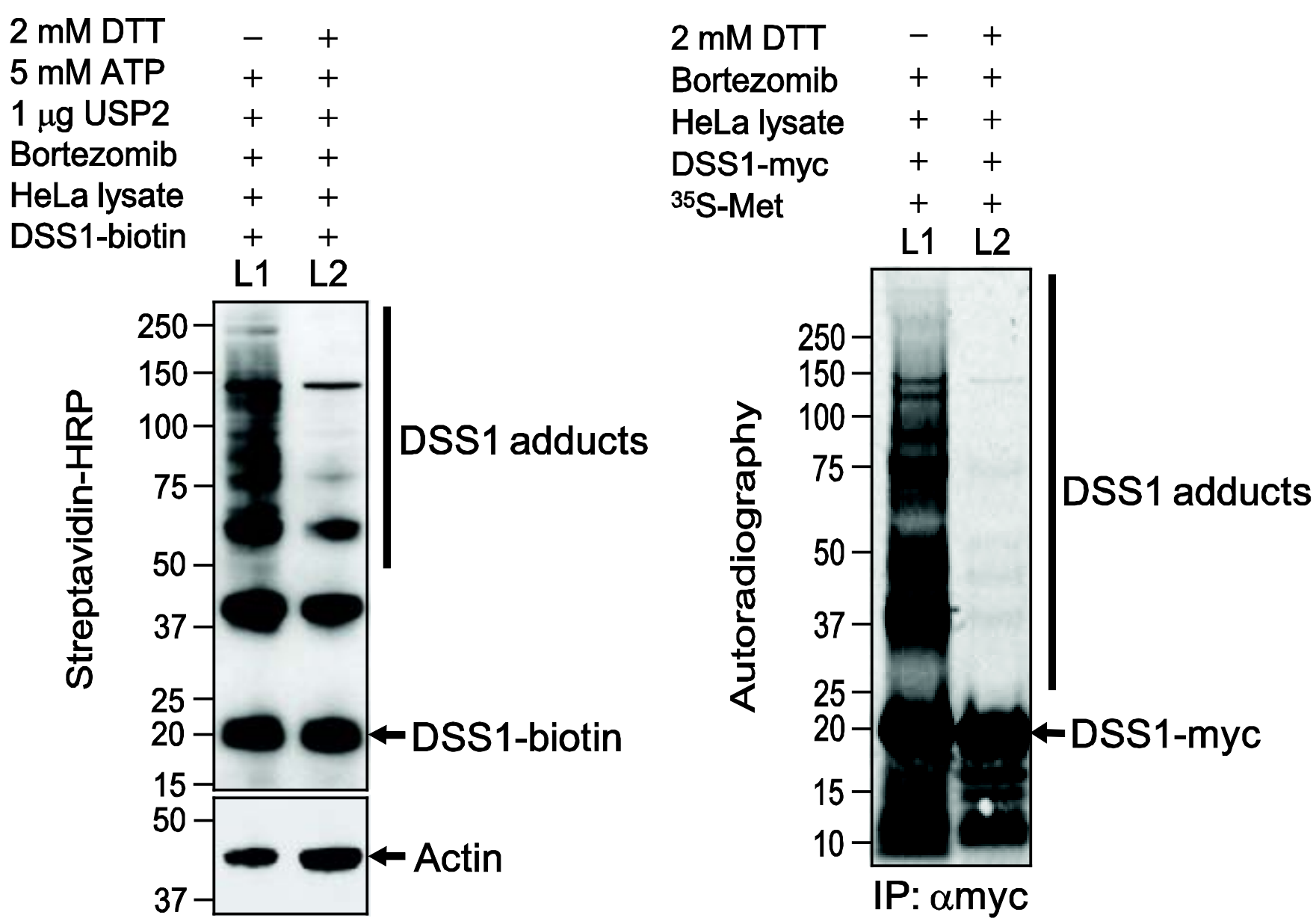

Figure S1. DTT blocks the formation of DSS1 conjugates.
DSS1-biotin (20 ng) or [^{35}S]-Met labeled DSS1-myc (20 µL) was incubated overnight at 4°C with HeLa lysate (50 µg) and Bortezomib (20 µmol/L) in the absence or presence of DTT (2 mmol/L), followed by WB or IP with anti-myc (40 µL) beads, and DSS1 and its adducts were identified by streptavidin-HRP (left panel) or developed by autoradiography (right panel). The membrane was stripped and re-probed with an anti-actin antibody and used as a loading control.

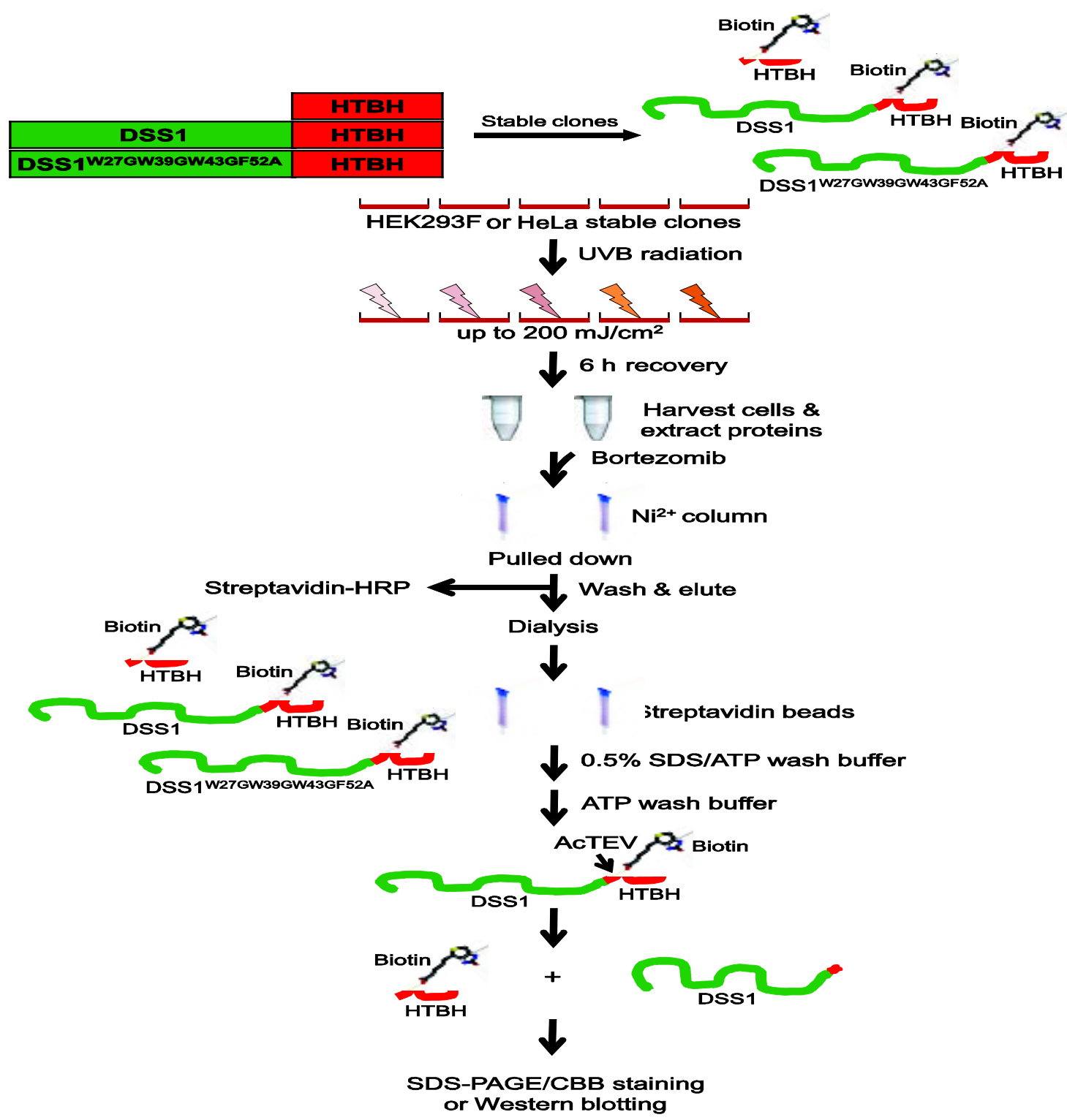

Figure S2. Flow chart for the UVB radiation treatment of the stable clones followed by lysate preparation and assessment of DSS1 adduct formation.

Retroviral vector pQCXIP, with or without an inserted human full-length *DSS1* cDNA, was fused with the *HTBH* gene at its COOH-terminus. RetroPack^{TM} PT-67 packaging cells were transfected with the pQCXIP harboring genes of interest including *HTBH*, *DSS1-HTBH*, and *DSS1*^{W27GW39GW43GF52A}*-HTBH* using lipofectAMINE®/PLUS^{TM} reagents. Virions were harvested and then transduced into HEK293F or HeLa cells to create *HTBH*, *DSS1-HTBH*, or *DSS1*^{W27GW39GW43GF52A}*-HTBH* stable cell clones. The infected cells were then incubated for 14 to 21 days under puromycin (1 µg/mL) selection, and two drug-resistant pooled clones, called HTBH-mix and DSS1-HTBH-mix, respectively, were chosen (from >100 resistant colonies). In addition, six additional clones were isolated that stably expressed *DSS1-HTBH* (four, designated as DSS1-HTBH-C2, -C9, -C14, and -C19) or *HTBH* alone (two, named HTBH-C14 and -C18). Four other DSS1 stable cell clones with mutations at amino acid residues W27, W39, W43, and F52 (DSS1^{W27GW39GW43GF52A}-HTBH-C1, -C2, -C7, and -C8) were also obtained for further studies. These cell clones were exposed to UVB radiation at doses ranging from 40 to 200 mJ/cm^2. After a 6 h period of recovery, cells were harvested for protein extraction in ATP lysis buffer in the presence of Bortezomib (20 µmol/L). Total protein lysates were first pulled down by Ni-NTA nickel beads, washed with ATP wash buffer, eluted with 350 mmol/L imidazole solution, and then dialyzed against 1× PBS using Slide-A-Lyzer dialysis cassettes with a 3,500 Da MWCO pore size (Pierce). Biotin-labeled HTBH or DSS1-HTBH and its conjugates were detected with streptavidin-HRP or bound to streptavidin agarose beads at 4°C overnight, and then enriched by washing with 0.5% SDS in ATP wash buffer. AcTEV was added to the mixtures followed by incubation with DSS1 and its conjugates at 30°C for 1 h to cleave the HTBH tag. DSS1 and its conjugates were separated by SDS-PAGE and detected by staining with CBB-R250 solution or WB with indicated antibodies.

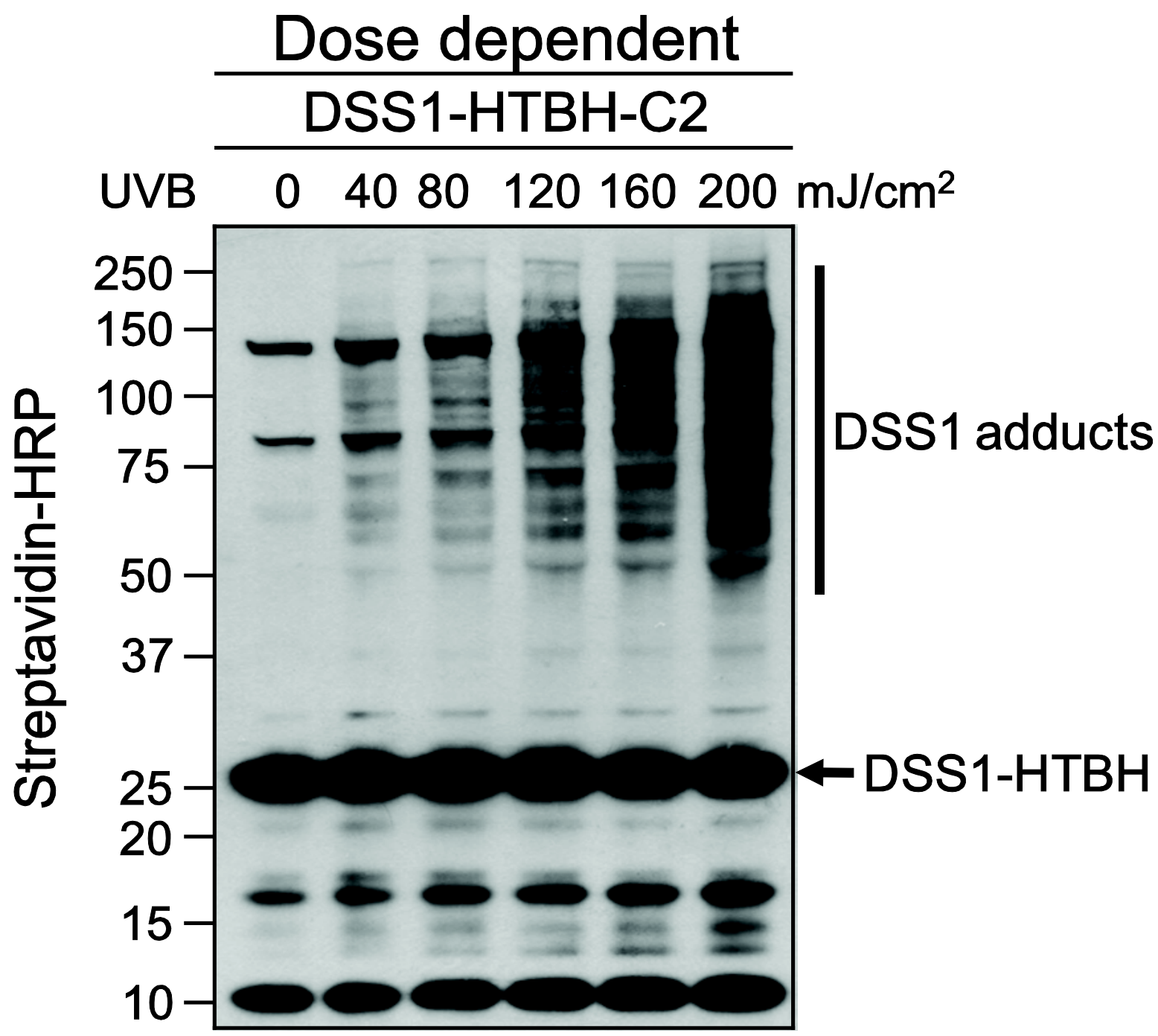

Figure S3. UVB radiation causes DSS1 conjugation in a dose-dependent manner in HEK293F cells.

Following exposure to UVB radiation, HEK293F/DSS1-HTBH-C2 cells were harvested as indicated. Total protein lysates (3 mg) were purified by Ni-NTA beads, and then DSS1 and its conjugates were detected with streptavidin-HRP.

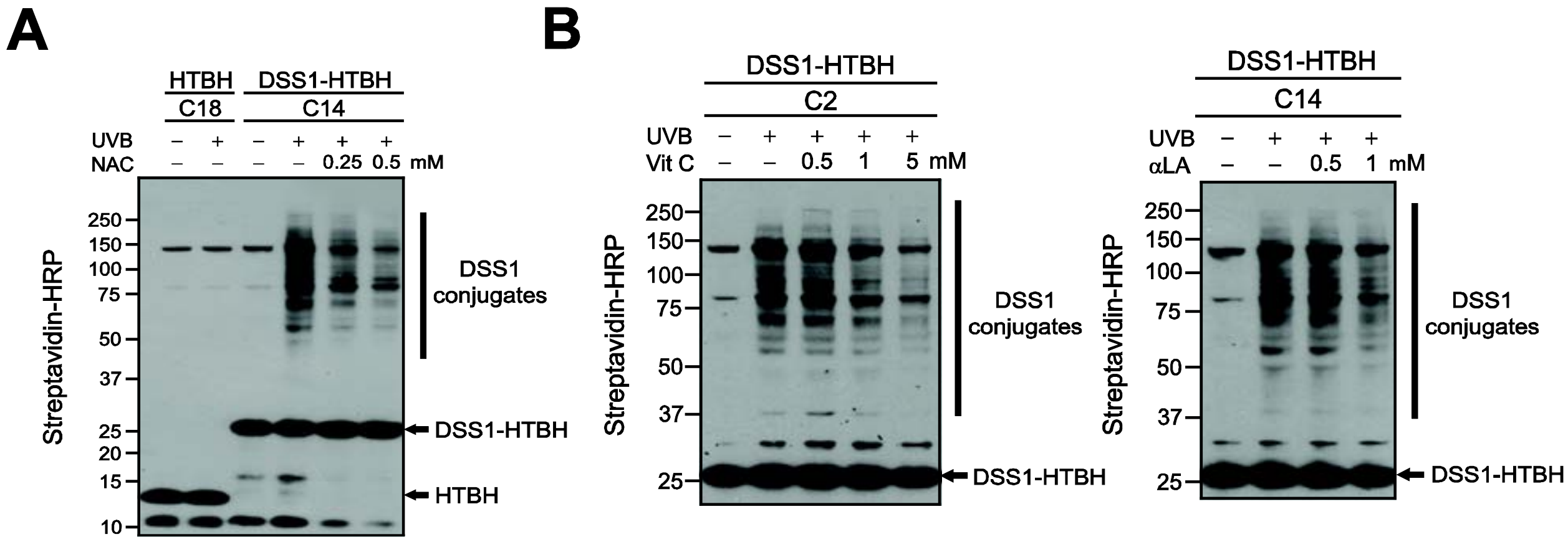

Figure S4. Antioxidants abrogate DSSylation in DSS1 sensor cells subjected to UV radiation.

(A) NAC inhibits UVB radiation-induced DSSylation. HEK293F/HTBH-C18 or HEK293F/DSS1-HTBH-C14 cells were treated with or without various doses of NAC for 24 h followed by irradiation with UVB at dose of 160 mJ/cm^2. After 6-hour recovery, DSS1 and its conjugates were pulled down by Ni-NTA beads and detected with streptavidin-HRP. (B) Vit C and αLA reduce UVB radiation-induced DSSylation in a dose-dependent manner in cells. HEK293F/DSS1-HTBH-C2 or HEK293F/DSS1-HTBH-C14 cells were treated with or without various doses of Vit C (24 h) or αLA (24 h) followed by irradiation with 160 mJ/cm^2 UVB. Six hours later, the experiments were performed as described above.

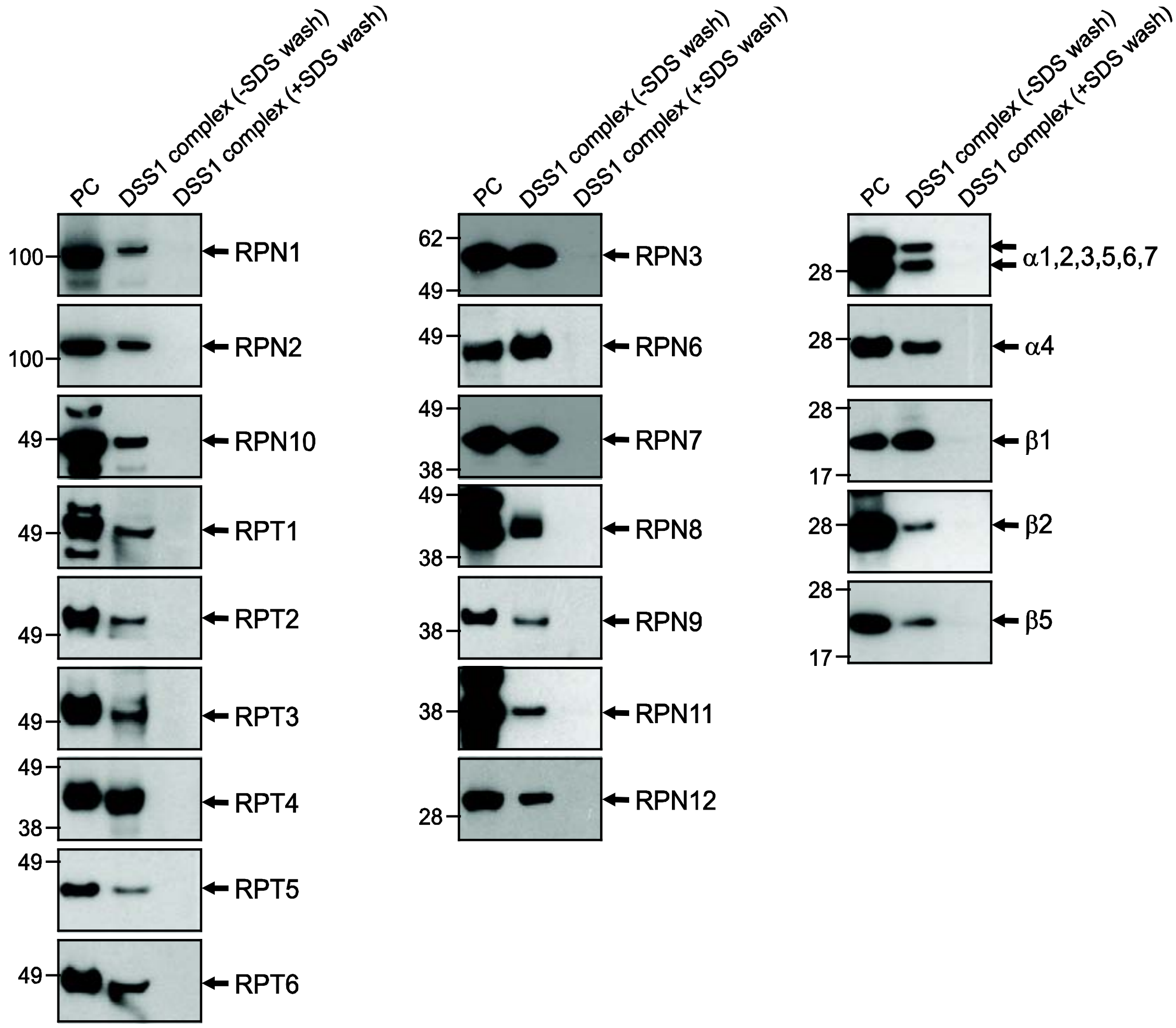

Figure S5. DSS1 binding partners were washed off from streptavidin agarose beads by 0.5% SDS in ATP wash buffer.
Six hours after exposure to UVB at 160 mJ/cm^2, total protein lysates extracted from HEK293F/DSS1-HTBH-C14 cells were prepared for pull-down assays in ATP lysis buffer, as described in Fig. S2. PC: 20 μg total protein lysate was used as a positive control; DSS1 complex (- SDS wash): total protein lysates were pulled down by Ni-NTA beads, washed with 4× ATP wash buffer, eluted by 350 mmol/L imidazole, and then dialyzed overnight against 1× PBS. The biotin-labeled DSS1-HTBH complex (with its binding partners) was incubated with streptavidin agarose at 4°C overnight and then washed with 4× ATP wash buffer. AcTEV was added to the mixtures and incubated at 30°C for 1 h to cleave the HTBH tag; DSS1 complex (+ SDS wash): the eluates from Ni-NTA pull-down were bound to streptavidin agarose beads at 4°C overnight and then washed with 4× 0.5% SDS (in ATP wash buffer). The DSS1-HTBH complex (without its binding partners) was digested with AcTEV to remove the HTBH. Both eluates were subjected to SDS-PAGE and detected by WB with the antibodies indicated.

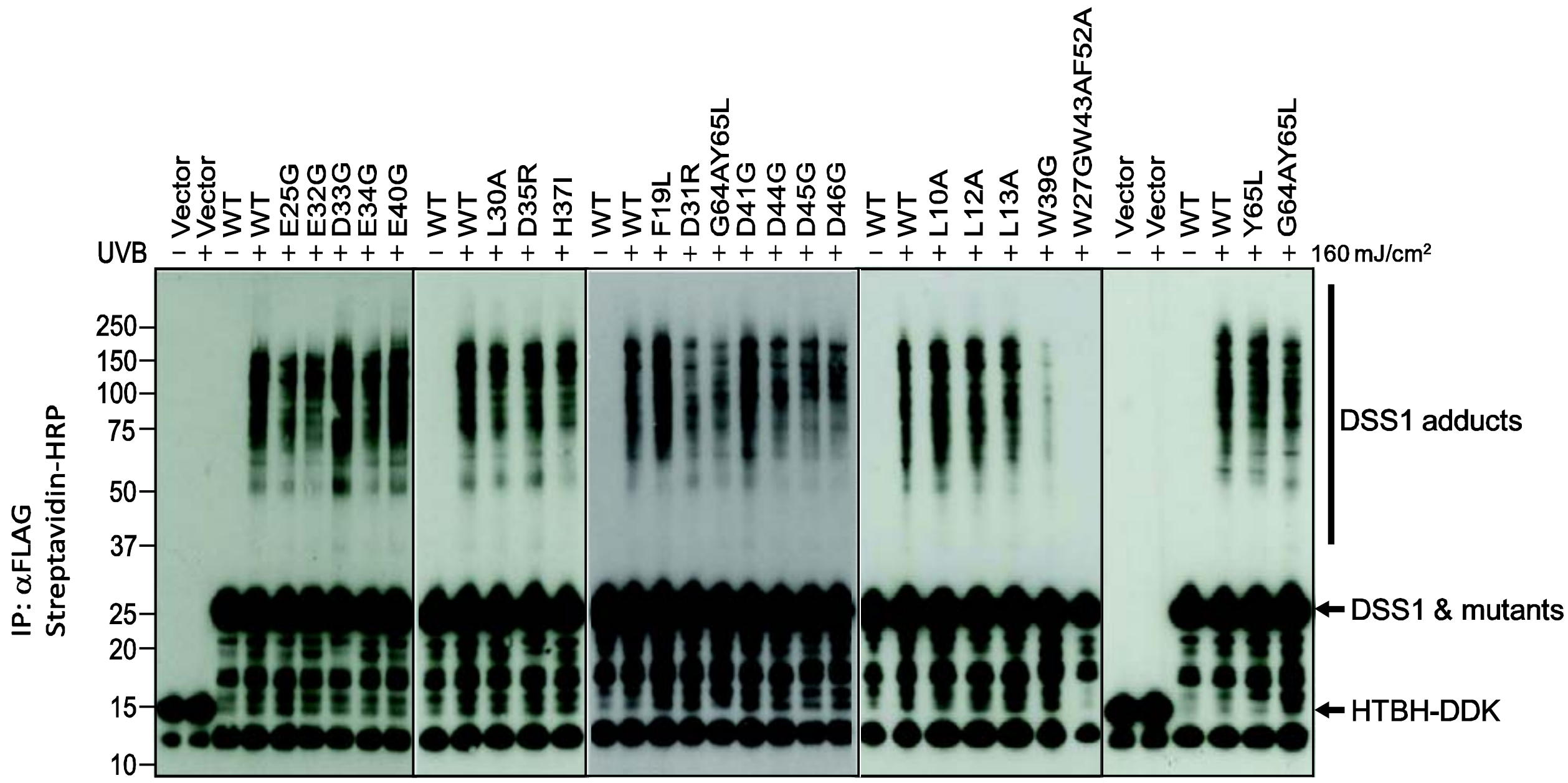

Figure S6. Screening crucial amino acid residue(s) that affect UVB radiation-induced formation of DSS1-protein adducts in DSS1 mutants.

Human wild-type *DSS1-HTBH-DDK* or its mutant genes were transiently expressed in HEK293F cells for 42 h. Six hours after treatment with or without UVB at 160 mJ/cm^2, total protein lysates (3 mg) were prepared for IP with anti-FLAG M2 affinity gel, and then DSS1 and its conjugates were identified by streptavidin-HRP. As compared to human wild-type DSS1, there was no significant change in the UVB radiation-induced formation of DSS1-protein adducts among the DSS1 mutants examined (L10A, L12A, L13A, E25G, L30A, D31R, E32G, E34G, D35R, H37I, E40G, D44G, D45G, D46G, Y65L, and G64AY65L). Unexpectedly, three DSS1 mutants with point mutations at amino acid residues F19, D33, and D41 were able to form more DSS1 conjugates than human wild-type DSS1-HTBH-DDK after exposure to UVB radiation.

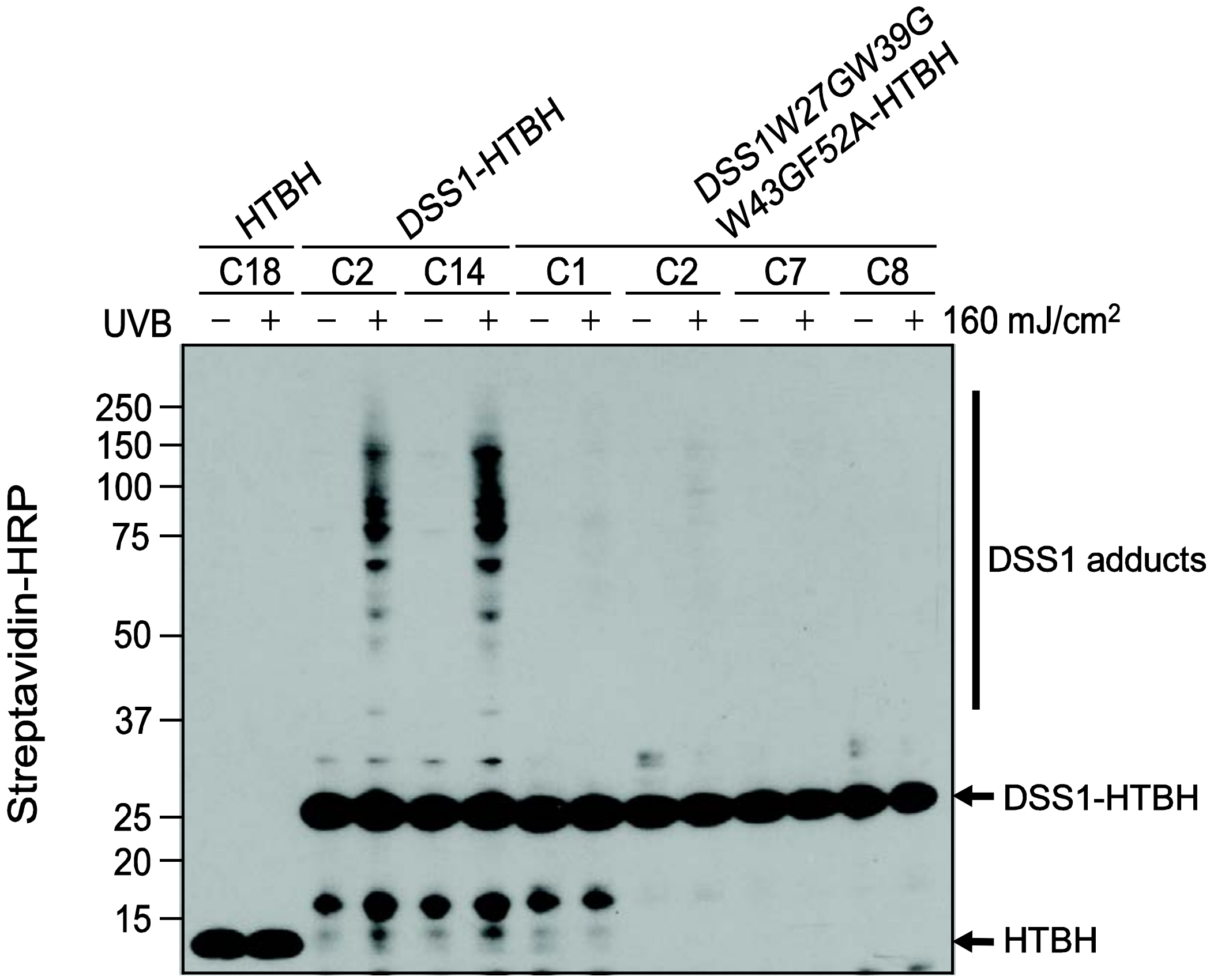

Figure S7. Physiological expression of a human *DSS1* gene with mutations at W27, W39, W43, and F52 in HEK293F cells abolished the UVB radiation-induced formation of DSS1-protein adducts.

HTBH (C18), DSS1-HTBH (C2 and C14) and $DSS1^{W27GW39GW43GF52A}$-HTBH (C1, C2, C7, and C8) stable cell clones were irradiated with or without 160 mJ/cm^2 UVB. Six hours later, total protein lysates (3 mg) were pulled down by Ni-NTA beads (100 μL) and HTBH, DSS1, and its conjugates were detected with streptavidin-HRP.
